# Idiopathic non-cirrhotic portal hypertension: A case report

**DOI:** 10.1097/MD.0000000000040642

**Published:** 2024-12-20

**Authors:** Qilong Nie, Qiuyan Liang, Mingyang Li, Ronghuo Zhu, Jian Ren, Kaiping Jiang, Jianhong Li

**Affiliations:** a The Eighth Clinical Medical College, Guangzhou University of Chinese Medicine, Foshan, Guangdong, China; b Foshan Hospital of Traditional Chinese Medicine, Guangzhou University of Chinese Medicine, Foshan, Guangdong, China.

**Keywords:** case report, hypercoagulable states, idiopathic non-cirrhotic portal hypertension, liver biopsy, portal hypertension

## Abstract

**Rationale:**

Idiopathic noncirrhotic portal hypertension (INCPH) is a rare liver disorder with elevated portal pressure without cirrhosis, making diagnosis challenging. This case report presents a 46-year-old Chinese male with INCPH, highlighting the crucial role of liver biopsy.

**Patient concerns:**

A 46-year-old male presented with persistent fatigue that lasted for 2 months and significantly worsened over the last 3 days. The patient described his fatigue as a profound lack of energy that persisted throughout the day, which progressively impaired his ability to perform daily activities and maintain his usual work responsibilities. He reported feeling exhausted even after light physical exertion, such as walking or standing for short periods. The severity of the fatigue also led to frequent short rests during the day, and he experienced difficulty concentrating and carrying out routine tasks. In addition, he noted a loss of appetite and mild discomfort in the upper abdomen. Given his previous history of abnormal liver function tests and a liver biopsy showing mild chronic liver damage, the patient was initially diagnosed with cirrhosis at a local hospital. This initial diagnosis caused significant emotional distress, as the patient experienced a state of panic and anxiety over the implications of having a progressive liver disease. The psychological burden was evident in his reported difficulty sleeping and persistent worry about his health and future.

**Diagnoses:**

Initial imaging suggested portal hypertension and cirrhosis, but a liver biopsy ruled out cirrhotic changes, confirming INCPH by excluding other causes such as chronic hepatitis.

**Interventions:**

The patient received symptomatic treatment (acid suppression, gastric and liver protection) and underwent a liver biopsy. Histological analysis confirmed INCPH, ruling out cirrhosis.

**Outcomes:**

After the definitive diagnosis, the patient’s anxiety lessened. Fatigue and weakness improved with ongoing symptomatic treatment, and psychological support enhanced his overall well-being. His follow-up plan includes regular liver function monitoring, imaging for portal pressure changes, and potential anticoagulation therapy for thrombosis risks.

**Lessons:**

This case highlights the diagnostic difficulty of INCPH and underscores the importance of liver biopsy. Further research is needed to develop specific diagnostic tools and treatments for INCPH.

## 1. Introduction

Idiopathic Non-Cirrhotic Portal Hypertension (INCPH) is a rare liver disorder characterized by increased portal pressure in the absence of cirrhosis. INCPH is part of a broader spectrum of non-cirrhotic causes of portal hypertension, which include conditions such as PVT, hepatic schistosomiasis, and congenital hepatic fibrosis. However, in cases where the etiology remains unclear, these are collectively referred to as INCPH.^[1]^

The prevalence of INCPH varies widely between regions, being more common in developing countries with lower socioeconomic levels. In Japan and India, INCPH accounts for approximately 30% to 40% of all NCPH cases, while in Western countries, this proportion is significantly lower, ranging from 3% to 5%. Gender differences in the prevalence of INCPH have also been reported. For instance, the condition is slightly more common in men in Western countries and India, whereas in Japan, it predominantly affects women. Regarding the age of onset, the median age in Western countries is around 40 years, while in Japan it ranges from 43 to 56 years, and in India from 25 to 35 years. Additionally, INCPH can occur in children, further complicating its demographic profile.^[2,3]^

The primary diagnostic challenge associated with INCPH arises from its clinical similarities with cirrhotic portal hypertension. Patients with INCPH often present with nonspecific symptoms, such as splenomegaly, esophagogastric varices, and portal hypertension, which are also hallmark features of cirrhosis. Conventional imaging modalities like ultrasound and magnetic resonance imaging (MRI) may indicate portal hypertension but often fail to reveal the subtle histological differences between cirrhotic and non-cirrhotic etiologies. As a result, many patients with INCPH are initially misdiagnosed with cirrhosis, leading to inappropriate management and missed therapeutic opportunities.

To address these challenges, Gottardi et al proposed a new classification term, “Portal Sinusoidal Vascular Disease” (PSVD), to describe a subgroup of NCPH that primarily affects the portal and sinusoidal vasculature.^[4]^ This new terminology is significant as it emphasizes the role of vascular pathology, guiding clinicians to consider INCPH in the differential diagnosis of patients presenting with unexplained portal hypertension. By focusing on specific histopathological changes within the portal and sinusoidal systems, the PSVD classification aims to improve diagnostic accuracy and patient outcomes.

## 2. Case presentation

A 46-year-old male was admitted to our hospital on February 25, 2024, with the chief complaint of “fatigue for 2 months, worsening over 3 days.” The patient’s clinical course is summarized in chronological order (Table 1).

**Table 1:** Chronological summary of patient’s clinical course.

Date	Event/observation
2011	First experienced fatigue and underwent liver biopsy, which revealed chronic hepatocellular damage with mild fibrosis. Regular follow-up initiated.
December 2023	Experienced worsening fatigue, prompting hospitalization and initial evaluations at Fengjie County Hospital of Traditional Chinese Medicine.
February 20, 2024	Admitted to Fengjie County Hospital of Traditional Chinese Medicine. Imaging suggested portal hypertension and splenomegaly. Diagnosed with potential cirrhosis and discharged with a recommendation for further evaluation.
February 24, 2024	Discharged from Fengjie County Hospital with a recommendation to visit Foshan Hospital of Traditional Chinese Medicine for further assessment.
February 25, 2024	Admitted to Foshan Hospital of Traditional Chinese Medicine for comprehensive diagnostic evaluation. MRI and laboratory tests indicated a possible cirrhosis diagnosis.
February 28, 2024	Liver biopsy performed, revealing findings consistent with INCPH (idiopathic non-cirrhotic portal hypertension). Mild fibrosis noted, and previous 2011 biopsy findings reviewed.
March 2024 onwards	Symptomatic treatment continued with psychological counseling. Significant improvements in fatigue and sleep quality reported. Ongoing follow-up plan established.

MRI = magnetic resonance imaging.

### 2.1. Past medical history (2011–2023)

The patient first experienced fatigue in 2011 and underwent a liver biopsy at our hospital, which revealed chronic hepatocellular damage with mild fibrosis. Since then, the patient attended regular follow-up visits at our hospital, where a series of laboratory and imaging examinations were performed to monitor the progression of his liver condition and guide ongoing management.

Laboratory examination results of hepatitis B markers over this period are presented in Table 2, while liver function test results are shown in Table 3. Results from routine blood examinations, conducted during each visit, are outlined in Table 4. The patient’s ultrasound findings for the liver, bile, pancreas, and spleen are summarized in Table 5, providing insight into structural changes over time. Additionally, liver transient elastography findings, essential for assessing liver stiffness, are presented in Table 6.

**Table 2:** The laboratory examination of hepatitis B.

Items/date	October 4, 2011	May 7, 2013	March 19, 2018	February 26, 2024	Reference range
HBsAg (IU/mL)	0.001	0.521	0.01	<0.05	<0.08
HBsAb (mIU/mL)	158.26	109.9	45.44	28.88	<10.0
HBeAg (S/CO)	0.134	0.117	0.43	0.18	<1.0
HbeAb (S/CO)	0.07	0.737	0.62	1.05	>1.0
HBcAb (S/CO)	5.4	0.007	7.45	0.01	>1.0

**Table 3:** The laboratory examination of liver function.

Date/items	ALT (U/L)	AST (U/L)	GGT (U/L)	ALP (U/L)	TBIL (µmol/L)	DBIL (µmol/L)	IBIL (µmol/L)
February 26, 2024	29.2	31.6	10.5	68.2	12.8	2.63	10.17
December 5, 2023	13	23.6	8	65.5	21.29	6.83	14.46
December 28, 2022	11	21.4	10.5	71.5	12.58	4.63	7.95
April 6, 2022	13.6	21.1	14.5	81.6	21.82	6.66	15.16
March 18, 2021	11.7	20.4	7.5	73	22.87	7.65	15.22
June 25, 2020	10.5	19.7	11	68	17.86	5.89	11.97
March 15, 2020	23.8	27.1	14.9	80	21.3	6.95	14.35
October 5, 2018	12	19.1	10	64.7	12.69	4	8.69
August 6, 2018	10.7	19.8	12.7	65.6	23.18	7.82	15.36
January 18, 2018	16.5	21.8	10.6	53.7	18.97	6.62	12.35
November 6, 2017	12.1	17.2	10.5	59.9	9.93	3.33	6.6
October 5, 2017	7.6	16.2	9.5	54.9	8.11	3.11	5
October 28, 2016	19	27.5	8.1	49.3	19.94	8.19	11.75
February 21, 2016	31.4	41	12.1	54.2	15.64	6.5	9.14
November 1, 2015	14.8	20.7	14.2	47.8	17.32	8.17	9.15
July 11, 2015	17	25.7	11.1	54.5	25.58	11.58	14
February 11, 2015	19.8	22.3	14.7	56.2	18.06	5.48	12.58
October 2, 2014	18.4	22.5	9.6	65.2	19.63	7	12.63
May 1, 2014	20.6	25.8	5.9	67	19.63	6.21	13.42
October 23, 2013	13.9	17.4	13.5	72.5	19.69	4.9	14.79
July 28, 2013	19.7	23.5	14.8	76.8	25.43	8.35	17.07
June 11, 2013	16.4	30	8.3	75.5	24.2	7.38	16.82
May 1, 2013	15.6	31.2	11.5	78	12.64	2.85	9.79
January 4, 2013	19.1	22.5	11.5	65.4	12.47	3.54	8.93
August 14, 2012	13.4	30.1	10.8	74.6	19.67	6.27	13.4
December 9, 2011	14.7	19.4	138	68.6	17.16	6.12	11.04
October 2, 2011	16.3	21.5	11.3	70	20.19	7.37	12.82
Reference range	9–50	15–40	10–60	53–128	2.0–20.4	0–6.8	3.2–12.2

ALP = alkaline phosphatase, ALT = alanine transaminase, AST = aspartate transaminase, DBIL = direct bilirubin, GGT = gamma-glutamyl transferase, IBIL = indirect bilirubin, TBIL = total bilirubin.

**Table 4:** The laboratory examination of blood routine.

Items/date	December 9, 2011	May 7, 2013	May 1, 2014	November 1, 2015	February 1, 2016	March 15, 2020	December 28, 2022	November 11, 2023	February 26, 2024	Reference range
RBC (10*12/L)	4.94	4.90	6.11	5.29	4.95	4.43	5.13	5.27	4.54	4.3 to 5.8
HGB (g/L)	144.00	143.00	161.00	158.00	153.00	137.00	156.00	163.00	137.00	130 to 175
MCV (FL)	85.10	85.60	87.10	80.90	85.30	87.80	85.20	85.60	87.00	82 to 100
MCHC (g/L)	340.00	341.00	362.00	369.00	363.00	352.00	357.00	361.00	347.00	316 to 354
PLT (10*9/L)	171.00	142.00	147.00	151.00	101.00	111.00	144.00	192.00	152.00	125 to 350
NEU (10*9/L)	2.55	0.67	2.84	2.30	71.80	3.86	3.29	3.35	2.74	1.8 to 6.3
LYM (10*9/L)	1.17	0.23	1.50	1.07	20.60	1.96	20.10	1.26	1.29	1.1 to 3.2
EOS (10*9/L)	0.13	0.02	0.04	0.04	0..02	0.04	0.03	0.08	0.05	0.02 to 0.52

EOS = eosinophils, HGB = hemoglobin, LYM = lymphocytes, MCHC = mean corpuscular hemoglobin concentration, MCV = mean corpuscular volume, NEU = neutrophils, PVT = portal vein thrombosis, RBC = red blood cell.

**Table 5:** The summary of ultrasound results of liver, bile, pancreas and spleen.

Date	Liver, gallbladder, pancreas, spleen ultrasound
October 2, 2011	1. Heterogeneous liver parenchyma echotexture. 2. Slightly enlarged spleen (spleen thickness 45 mm, length 126 mm).
October 22, 2013	1. Slightly coarse liver parenchyma echotexture. 2. Spleen not reported as slightly enlarged (spleen thickness 45 mm, length 126 mm).
October 2, 2014	1. Spleen not reported as slightly enlarged. 2. Spleen not reported as slightly enlarged.
October 28, 2016	No abnormalities seen in the liver, spleen appears full-bodied.
March 5, 2020	1. No abnormalities observed in the liver. 2. Spleen slightly enlarged (spleen thickness 45 mm, length 126 mm, splenic vein width 5 mm).
March 23, 2021	1. No abnormalities observed in the liver. 2. Spleen slightly enlarged (spleen thickness 40 mm, length 122 mm, splenic vein width 7 mm).
December 4, 2023	1. Hepatic parenchymal nodule (A slightly hyperechoic nodule measuring 12 mm*10 mm observed in segment S5 of the liver, with clear boundaries, regular margins, uneven internal echoes, and rich blood supply, grade 0). 2. Spleen slightly enlarged (spleen thickness 29 mm, length 122 mm, splenic vein width 5 mm).

**Table 6:** The summary of liver transient elastography.

Date	August 14, 2012	January 4, 2013	May 2, 2013	January 18, 2018	August 6, 2018	April 6, 2022	November 11, 2023	January 2, 2024	February 27, 2024
LSM (KPA)	5	5.5	6.6	6.3	5.9	4.7	8.9	5.8	5.3

LSM (KPA) = Liver Stiffness Measurement (Kilopascals).

### 2.2. Initial presentation (December 2023 to February 2024)

In December 2023, the patient experienced worsening fatigue and was hospitalized at Fengjie County Hospital of Traditional Chinese Medicine on February 20, 2024. Ultrasound examination revealed small cysts in the liver and a slightly enlarged spleen (spleen thickness 37 mm, length 124 mm). Enhanced CT of the upper abdomen showed a thickened portal vein, a tortuous and thickened splenic vein, and splenomegaly, indicating portal hypertension. The patient received symptomatic treatment, including acid suppression, gastric protection, and liver protection. During this period, he was informed by the attending physicians of a potential diagnosis of cirrhosis based on imaging findings, which caused significant anxiety and panic. Given the complexity of the case and the suspicion of cirrhosis, the doctors at Fengjie County Hospital recommended that the patient seek further evaluation and treatment at a higher-level facility. The patient was discharged on February 24, 2024, and advised to proceed to Foshan Hospital of Traditional Chinese Medicine for further examination.

### 2.3. Further diagnosis (February 25, 2024)

**Figure 1.**
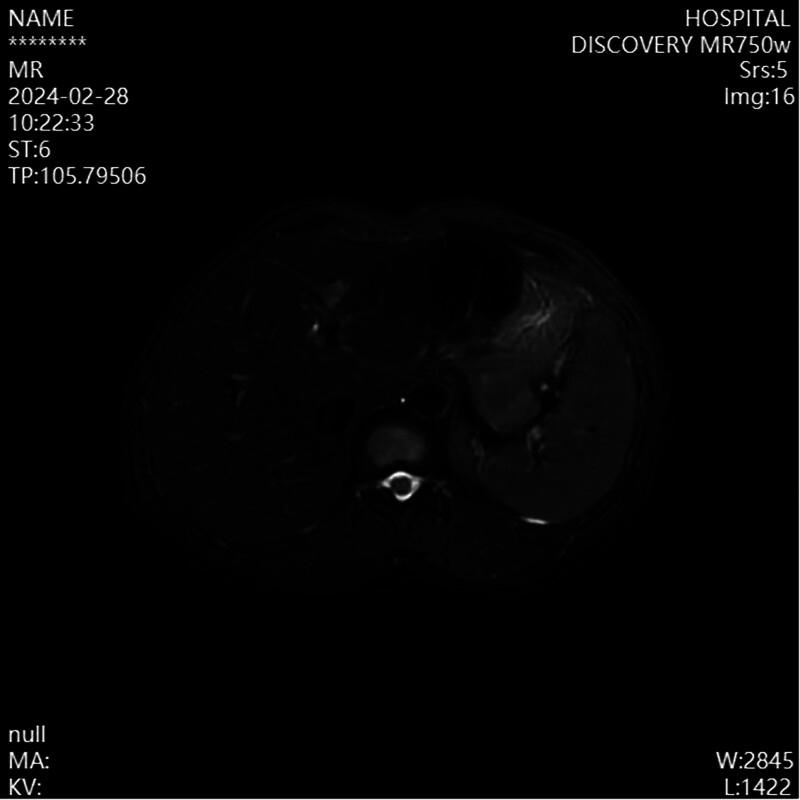
Enhanced MRI of the upper abdomen shows: the liver appears mildly irregular, the spleen is enlarged, with its lower edge significantly extending below the lower margin of the liver; the splenic vein is wide, and tortuous vascular shadows are seen at the splenic hilum. MRI = magnetic resonance imaging.

The patient was admitted to our hospital for comprehensive diagnostic evaluation. Laboratory tests, including blood routine, liver function, kidney function, coagulation profile, hepatitis B panel, and autoimmune liver disease antibody panel, all showed no abnormalities. Upper abdominal MRI findings indicated a high possibility of cirrhosis, with splenic and portal vein varices and splenomegaly (Fig. 1). Gastroscopy revealed no esophagogastric varices but showed chronic non-atrophic gastritis with bile reflux. Despite imaging results suggestive of cirrhosis, the patient displayed no clinical signs such as jaundice, spider nevi, or hepatic encephalopathy, prompting the medical team to consider further investigation.

### 2.4. Liver biopsy (February 28, 2024)

**Figure 2.**
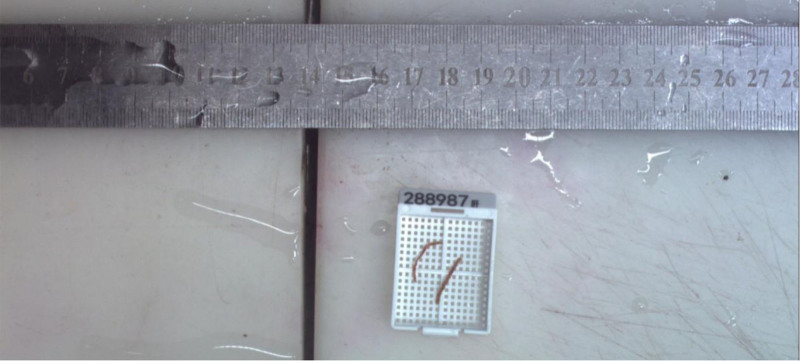
Liver biopsy tissue from 2 samples, each approximately 1.3 cm in length and 0.1 cm in diameter.

A liver biopsy was performed to establish a definitive diagnosis (Fig. 2). The histopathological examination of the liver biopsy revealed the following findings:

Liver lobule structure: Observed 14 portal areas, with overall preservation of lobular architecture.

**Figure 3.**
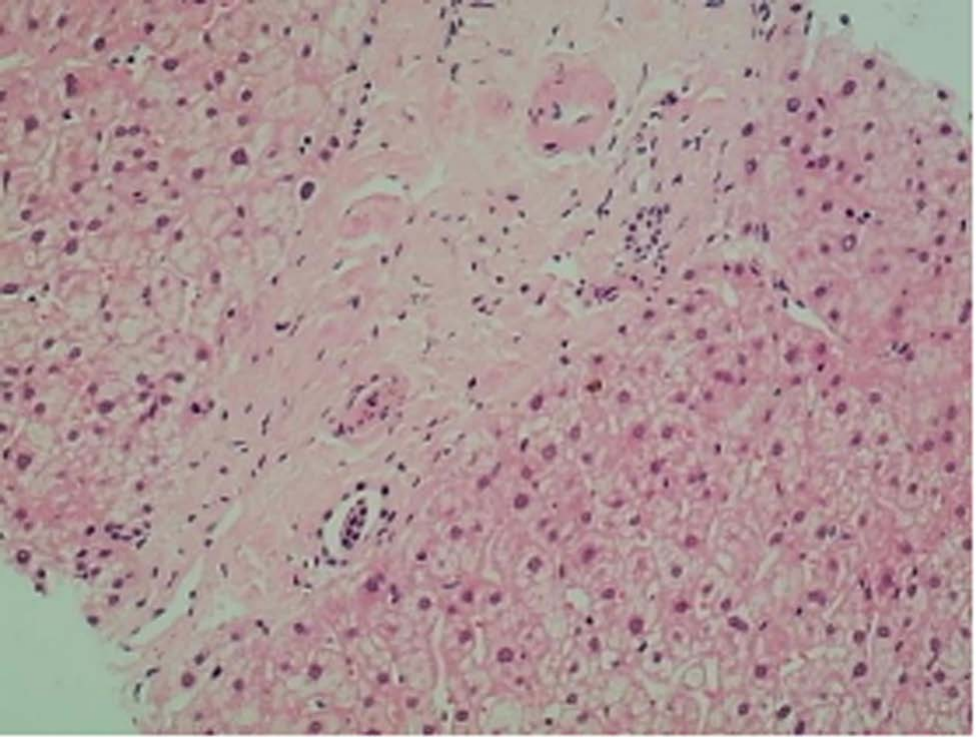
Fibrous expansion in the portal tracts, thickening of the walls of the interlobular arteries with luminal narrowing, and occlusion of the interlobular veins.

**Figure 4.**
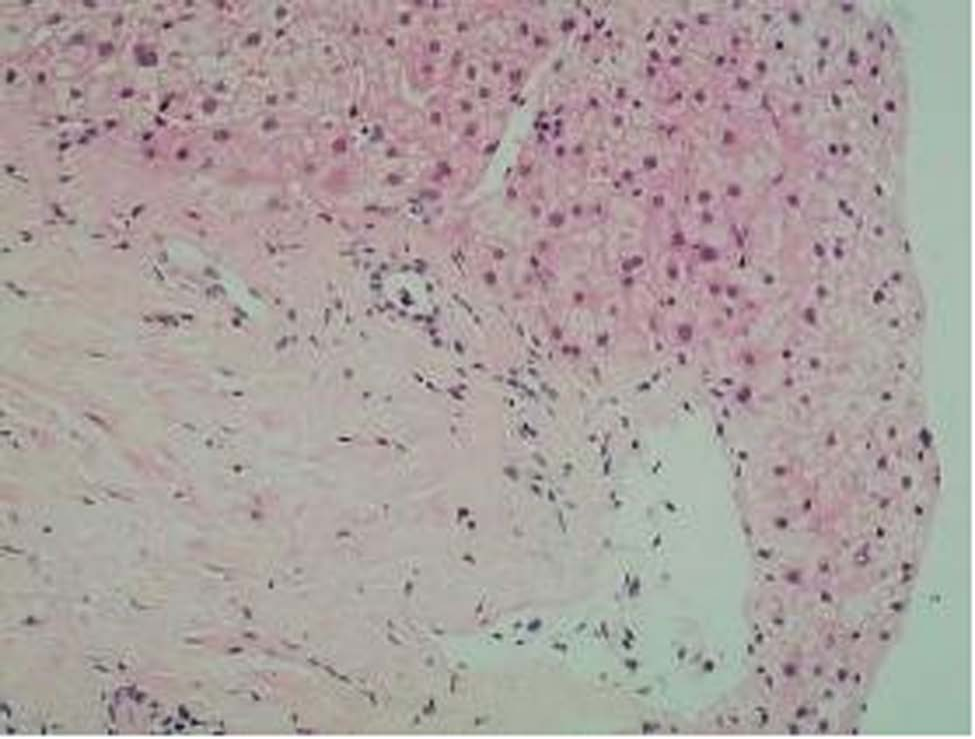
Pericentral venous fibrosis.

Portal areas: Several portal areas exhibited fibrous expansion with mild lymphocytic infiltration, but without interface hepatitis. The interlobular bile ducts showed mild narrowing, with evidence of mild fibrous tissue proliferation (Fig. 3). The walls of interlobular arteries were slightly thickened, and certain interlobular veins were highly dilated, with some branches herniating into the hepatic lobules. A few interlobular veins showed luminal narrowing (Fig. 4).

**Figure 5.**
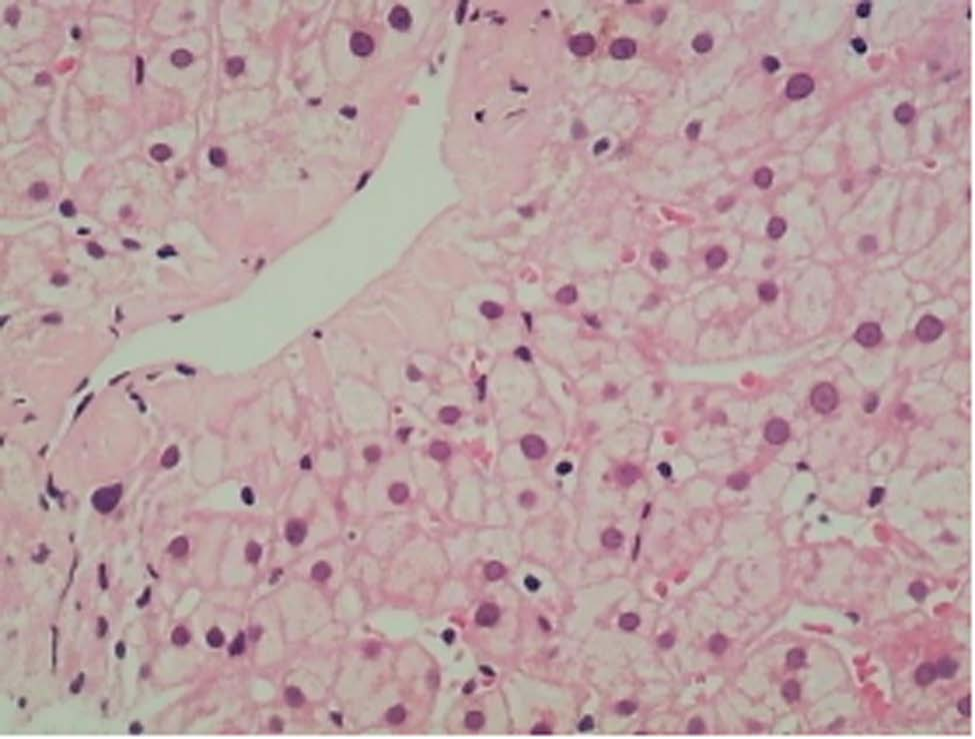
Perilobular venous fibrosis with branches herniating into the liver lobules.

Hepatocytes: Some hepatocytes exhibited edema, with occasional focal necrosis, but no significant fatty changes or Mallory bodies were observed. Hepatic sinusoids were not dilated, and Kupffer cells showed no proliferation. Some central veins were dilated, with evidence of pericentral venous fibrosis in a few areas (Fig. 5).

**Figure 6.**
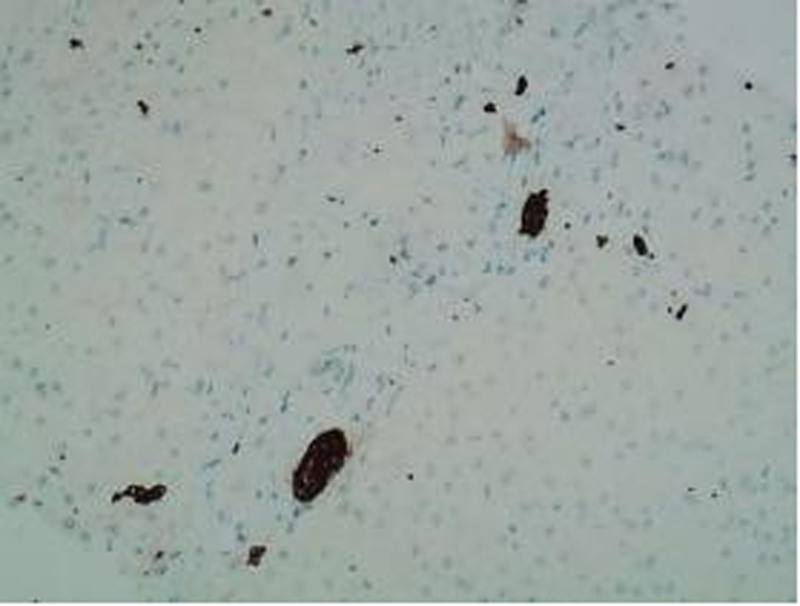
CK19 bile duct epithelium (+). CK19 = Cytokeratin 19.

Immunohistochemistry: CD68(−); SMA staining indicated activation of a few stellate cells; MUM-1(−); IgG(−); IgG4(−). CK7 and CK19 were positive in bile duct epithelium, demonstrating bile duct narrowing (Fig. 6).

**Figure 7.**
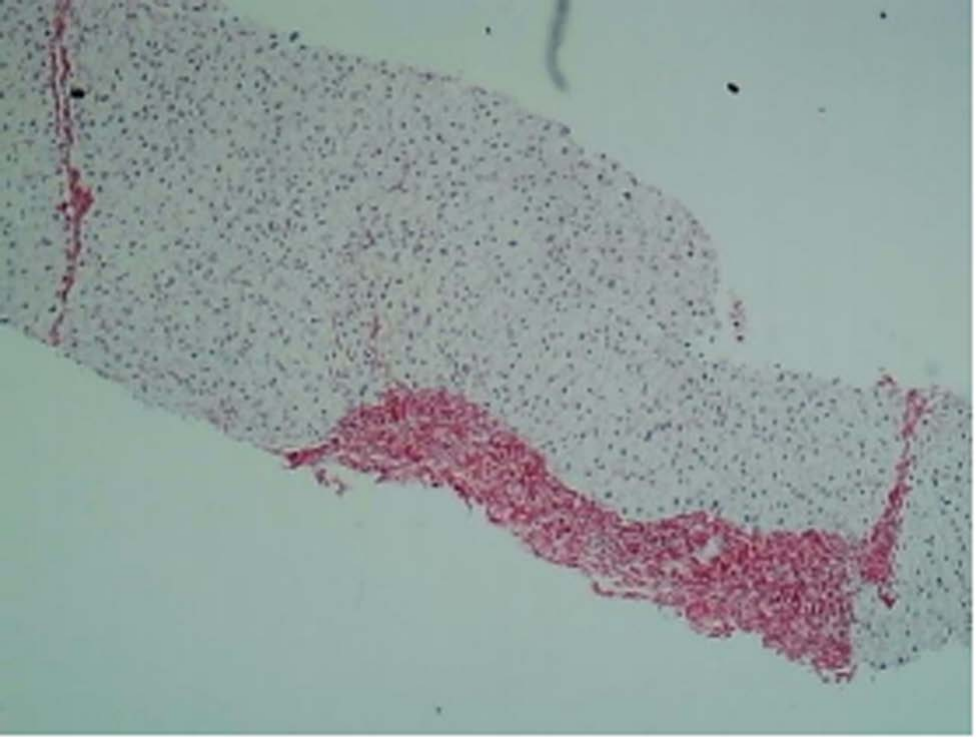
Sirius Red staining shows fibrous septa.

**Figure 8.**
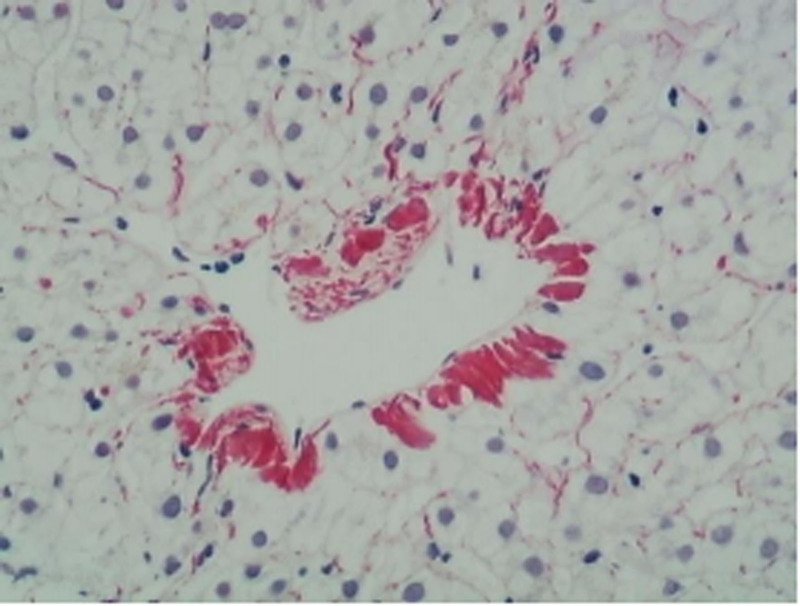
Sirius Red staining shows pericentral venous fibrosis.

Special stains:

Masson and Sirius Red: Showed mild fibrous tissue proliferation with a few stellate fibers and formation of 3 fibrous septa (Figs. 7 and 8).

Reticulin stain: No significant collapse of the hepatic reticular framework.

PAS stain: PAS(+) indicating no significant increase in hepatic glycogen content.

DPAS stain: Negative.

Iron stain: Negative.

Copper stain: Negative.

Diagnosis and recommendations: The findings were consistent with mild chronic liver damage, with a Scheuer score of G1S2. The report noted the presence of abnormal portal vein branches and suggested excluding conditions such as non-cirrhotic portal hypertension (NCPH) and PSVD. Compared with the previous liver biopsy performed in 2011, the current findings revealed increased interlobular vein narrowing and thickening of interlobular artery walls, along with more prominent fibrosis and bile duct narrowing.

### 2.5. Management and follow-up (March 2024 onwards)

The patient expressed considerable relief upon learning that his condition was not cirrhosis, which alleviated his previous anxiety and panic. Symptomatic treatment, including acid suppression and liver protection, was continued. Psychological counseling was also provided to help manage emotional distress. During the most recent follow-up, the patient reported significant improvements in fatigue and sleep quality. An ongoing follow-up plan was established, including:

Regular monitoring of LFTs and portal pressure assessments.

Periodic imaging evaluations, such as Doppler ultrasound, to assess portal vein and splenic conditions and screen for potential complications like PVT or splenic varices.

Lifestyle modifications, including a low-sodium diet and avoiding activities that may exacerbate portal pressure.

Consideration of anticoagulation therapy based on future risk assessments.

## 3. Follow-up and outcomes

A case of INCPH was admitted to our hospital. The patient, a middle-aged male, presented with fatigue and was found to have portal hypertension upon examination. Comprehensive biochemical tests and an abdominal MRI were performed, leading to a preliminary diagnosis of liver cirrhosis, splenomegaly with splenic portal vein varices, and multiple small cysts in the right hepatic lobe. A retrospective study of 388 cases of IPH in China demonstrated that the most common clinical manifestations of IPH are splenomegaly (91.3%) and hypersplenism (68.9%).^[5]^ To further clarify whether the cirrhosis has other complications, additional unexamined procedures were completed, and the results indicated no esophagogastric varices but chronic non-atrophic gastritis with bile reflux. To determine if the patient had cirrhosis, a liver biopsy was performed. Pathological examination revealed that the lobular architecture of the liver was mostly preserved, with no pseudo-lobules or nodular hepatocyte hyperplasia. There was mild fibrotic expansion in some portal areas with a few lymphocytic infiltrates and no interface hepatitis. The pathological findings ruled out the diagnosis of cirrhosis. Liver biopsy is essential for differential diagnosis and treatment planning, as imaging alone may not reveal detailed pathological changes. The characteristic portal vein lesions in INCPH include luminal narrowing, missing portal vein branches, and varying degrees of portal area fibrosis, accompanied by small bile duct proliferation. Additional pathological changes around the portal vein include herniation of the portal vein and hemangioma-like changes in the periportal areas.^[6–8]^ The primary manifestation of hepatic venous lesions is central vein dilation, with or without perivenous fibrosis. It has been reported that 44% of INCPH patients exhibit an abnormal increase in the number of central vein profiles.^[9]^ Histological examination of this patient’s liver tissue showed no pseudo-lobules or nodular hyperplasia, effectively ruling out cirrhosis. However, there was mild fibrotic expansion in some portal areas, no significant changes in the small bile ducts, mild proliferation of fibrous tissue, slight thickening of the interlobular artery walls, marked dilation of some interlobular veins, herniation of a few branches into the hepatic lobules, and narrowing of some interlobular vein lumina. Doppler ultrasound in INCPH patients often shows significant splenomegaly, dilation of the splenic portal vein, and thickening of the portal vein wall (>3 mm). In this patient, ultrasound indicated the portal vein main trunk with an internal diameter of approximately 12 mm, and the spleen measured 124 mm in length and 37 mm in thickness. Transient elastography of the liver can assess liver and spleen stiffness, which is very helpful in distinguishing between cirrhosis and INCPH.^[10,11]^ In patients with cirrhosis, liver stiffness is high while spleen stiffness is relatively low. Conversely, in patients with INCPH, spleen stiffness is high, and liver stiffness is relatively low. An Indian study demonstrated that liver stiffness in INCPH patients was significantly higher than in healthy controls (6.8 kPa vs 4.7 kPa, *P* = .001) but significantly lower than in the cirrhosis group (6.8 kPa vs 52.3 kPa, *P* = .001).^[12]^ This patient’s liver transient elastography result from February 27, 2021, was 5.3 kPa. The liver transient elastography results over the past ten years are shown in the table. The patient’s liver stiffness has consistently remained within the normal range, further ruling out cirrhosis. Based on the above tests and examinations, the final diagnosis is INCPH.

The patient will be followed up every 3 to 6 months to monitor the progression of INCPH and address any ongoing symptoms, particularly the persistence of fatigue. Each follow-up visit will include comprehensive assessments to ensure early detection and management of potential complications.

Given that fatigue remains a significant symptom for the patient, it will be regularly assessed using validated tools such as the Fatigue Severity Scale (FSS) or the Multidimensional Fatigue Inventory (MFI). The patient will be encouraged to maintain a symptom diary, documenting the severity, frequency, and impact of fatigue on daily life. This will allow the medical team to track changes in symptom burden and adjust the management plan accordingly. Considering the persistence of fatigue, the patient’s nutritional status will be regularly evaluated, and appropriate dietary modifications or supplements (e.g. vitamins, iron) will be recommended. Further exploration into contributing factors, such as sleep disturbances or emotional stress, will also be conducted. If the patient’s fatigue significantly affects quality of life, referral to a specialist in chronic fatigue or a multidisciplinary team (including a dietitian, psychologist, or physical therapist) may be considered.

Routine biochemical tests, including liver function tests (LFTs), complete blood count (CBC), and coagulation profiles, will be performed at each visit. Additionally, Doppler ultrasound will be used to monitor portal vein diameter, flow, and the presence of varices or thrombosis. If indicated, transient elastography will be conducted to assess liver stiffness and monitor disease progression.

Based on follow-up findings, adjustments to the patient’s treatment plan will be considered. For instance, if evidence of PVT is detected, anticoagulation therapy may be initiated or modified. Additionally, any new or worsening symptoms, including the exacerbation of fatigue, will prompt a reevaluation of supportive treatments, with possible interventions including physical therapy, lifestyle adjustments, or medications aimed at symptom relief.

The patient will be provided with ongoing education regarding lifestyle modifications to manage fatigue, including recommendations on physical activity, rest periods, and stress management. The importance of adhering to follow-up appointments, maintaining a healthy diet, and engaging in regular physical activity will be emphasized to help manage both INCPH and fatigue.

## 4. Discussion

As of now, there is no unified global guideline for the diagnosis of INCPH. Currently, there are no specific diagnostic tools for NCPH; the diagnosis primarily relies on exclusion. Diagnostic criteria for this condition have been proposed by Japanese scholars, the Asia-Pacific Liver Research Association, and Schouten et al Key elements for diagnosis include: Clinical evidence of portal hypertension, such as splenomegaly, esophagogastric varices, anemia, and thrombocytopenia. Early-stage patients may only present with recurrent unexplained liver function abnormalities; Exclusion of chronic hepatitis and cirrhosis; Exclusion of other causes of NCPH such as PVT, Budd–Chiari syndrome, and congenital hepatic fibrosis; and histological characteristics of INCPH.^[13]^

The etiology of INCPH remains unclear, but current perspectives suggest associations with 5 major factors: immune dysregulation, hypercoagulable states, chronic infections, exposure to drugs and toxins, and genetic disorders. Specifically, immune dysregulation: The development of INCPH is linked to immune dysregulation, as seen in conditions such as rheumatoid arthritis, systemic lupus erythematosus, systemic sclerosis, and scleroderma.^[14,15]^ The underlying mechanisms are currently unclear and require further investigation; Regarding hypercoagulable states: A study based on a cohort of 28 patients in France revealed that 50% of the patients exhibited prothrombotic disorders, including myeloproliferative neoplasms, deficiencies in protein S or C, and the presence of antiphospholipid antibodies.^[16]^ Similarly, a study from Turkey reported that 7 out of 34 patients with INCPH (20%) developed extrahepatic PVT during a 5-year follow-up period.^[17]^ Patients with hypercoagulable states have a higher incidence of INCPH, and those with INCPH are at a relatively higher risk of developing PVT. Additionally, liver biopsies in INCPH patients show obliterative portal venopathy, suggesting that thrombotic events in portal vein branches may be an anatomical basis for the development of INCPH. Consequently, anticoagulation therapy has been proposed as a treatment method for patients with INCPH; Chronic infections: The prevalence of INCPH is higher in regions with lower socioeconomic status, which may be linked to an increased likelihood of intra-abdominal infections. Studies indicate that gastrointestinal infections can lead to septic emboli that obstruct small portal veins, thereby triggering INCPH. Furthermore, HIV infection is also associated with INCPH; reports from India indicate that 1% of HIV-infected individuals have concurrent INCPH. The mechanisms by which HIV induces INCPH are not yet clear but may include increased risk of intestinal bacterial infections, direct viral effects, or immune-mediated damage^[18]^; Drug and toxin exposure: Medications, chemicals, and toxins are also associated with the incidence of INCPH, with azathioprine, oxaliplatin, and arsenic poisoning being the most common^[19,20]^; Genetic disorders: The familial clustering of INCPH and its association with certain congenital diseases, such as Adams-Oliver syndrome, Turner syndrome, and cystic fibrosis, suggest a genetic predisposition to the condition.^[21,22]^

The management of INCPH requires a comprehensive, multidisciplinary approach, given the chronic nature of the disease and its potential complications, such as splenomegaly, variceal bleeding, and PVT. Without appropriate management, these complications can severely affect patient outcomes. Therefore, long-term treatment strategies should focus on both symptomatic relief and the prevention of disease progression, with close monitoring of portal pressures and liver function.

A major consideration in the management of INCPH is whether patients should receive early anticoagulation therapy. This remains a subject of debate. A recent retrospective study indicated that anticoagulation in INCPH patients with portal vein thrombosis (PVT) led to partial recanalization in 54% of patients by the end of follow-up.^[23]^ Furthermore, early anticoagulation in hypercoagulable INCPH patients has been associated with improved prognosis. Low-molecular-weight heparin and vitamin K antagonists are the most commonly used agents in managing PVT, although emerging evidence suggests that newer oral anticoagulants may offer a safer and more effective option for treating PVT.^[24]^ Despite these promising results, anticoagulation therapy carries a significant risk of bleeding, necessitating a careful evaluation of the indications, therapeutic regimen, and potential benefits in preventing thrombosis and disease progression. The decision to initiate anticoagulation must therefore be individualized, factoring in the patient’s overall risk profile, potential bleeding complications, and the need for vigilant monitoring of coagulation parameters.

For the management of variceal bleeding in INCPH, endoscopic interventions play a central role. Endoscopic variceal ligation (EVL) and sclerotherapy (EST), especially when combined with vasoactive drugs like somatostatin, octreotide, or terlipressin, are effective in controlling acute bleeding and preventing recurrence. These therapies help manage 1 of the most serious complications of INCPH, ensuring reduced variceal pressure and rebleeding rates.^[25–28]^

In cases where endoscopic therapy fails or in the presence of severe complications like hypersplenism, shunt procedures become necessary. nonselective shunts, such as the distal splenorenal shunt (DSRS), and physiological shunts like the Rex shunt, offer effective decompression of the portal system while maintaining liver function. These surgeries have been shown to significantly reduce portal pressure and improve quality of life. For patients with hypersplenism, splenectomy or embolization may be required to alleviate symptoms and reduce splenic congestion.^[29,30]^

Long-term management of INCPH involves not only addressing acute complications but also continuous monitoring of disease progression. The use of liver-protective agents and regular assessment of liver function and portal pressures are essential in minimizing disease progression. The overall goal is to maintain patient well-being by tailoring the treatment approach to individual needs, adjusting interventions as necessary to prevent complications and manage portal hypertension effectively.

INCPH is a rare and complex condition often misdiagnosed clinically due to the lack of unified diagnostic standards and unidentified etiology. In this case, the patient was diagnosed with INCPH after detailed medical history, physical examination, and a series of auxiliary tests, which excluded cirrhosis and other common causes of portal hypertension. Liver biopsy played a crucial role in confirming the diagnosis, revealing typical pathological changes. This study highlights the diversity of etiologies associated with INCPH, including immune dysregulation, hypercoagulable states, chronic infections, exposure to drugs and toxins, and genetic factors. The importance of liver biopsy is emphasized, serving as an essential tool for definitive diagnosis and formulating treatment plans. Although there are no specific treatments for INCPH, anticoagulation therapy and comprehensive treatment targeting the underlying causes may improve patient prognosis. Overall, the diagnosis and management of INCPH require a multidisciplinary approach, utilizing exclusion diagnostics and histopathological examination to enhance diagnostic accuracy and develop individualized treatment strategies, ultimately improving patient quality of life.

## 5. Patient perspective

During a recent follow-up visit, the patient shared his experience regarding his diagnosis and treatment journey. He initially presented with fatigue and was misdiagnosed with liver cirrhosis at a local hospital, which caused him considerable anxiety. The thought of having cirrhosis and the potential complications, such as variceal bleeding and long-term liver damage, weighed heavily on his mind. After undergoing a series of diagnostic tests, including a liver biopsy that ruled out cirrhosis and confirmed INCPH, the patient expressed a sense of relief. Although INCPH is a chronic condition, he felt reassured by the fact that his liver’s structure remained largely intact and that there were no signs of severe liver damage. He emphasized how the detailed diagnostic process helped alleviate his initial fears. Looking forward, the patient emphasized the importance of regular monitoring and expressed confidence in the current management plan. While there is no specific curative treatment, he appreciated that ongoing follow-up and monitoring of his liver function and portal pressures gave him a sense of control and peace of mind.

## Author contributions

**Conceptualization:** Qilong Nie, Qiuyan Liang, Mingyang Li, Ronghuo Zhu, Jian Ren.

**Funding acquisition:** Jianhong Li.

**Supervision:** Jianhong Li.

**Visualization:** Qilong Nie, Qiuyan Liang, Mingyang Li.

**Writing – original draft:** Qilong Nie.

**Writing – review & editing:** Kaiping Jiang, Jianhong Li.
